# Personal Glucose Meter for α-Glucosidase Inhibitor Screening Based on the Hydrolysis of Maltose

**DOI:** 10.3390/molecules26154638

**Published:** 2021-07-30

**Authors:** Tao Tian, Guo-Ying Chen, Hao Zhang, Feng-Qing Yang

**Affiliations:** 1 School of Chemistry and Chemical Engineering, Chongqing University, Chongqing 401331, China; 202018021153t@cqu.edu.cn (T.T.); 201918021143@cqu.edu.cn (G.-Y.C.); 2 Chongqing Key Laboratory of High Active Traditional Chinese Drug Delivery System, Chongqing Medical and Pharmaceutical College, Chongqing 401331, China

**Keywords:** α-glucosidase, personal glucose meter, inhibitor screening, molecular docking

## Abstract

As a key enzyme regulating postprandial blood glucose, α-Glucosidase is considered to be an effective target for the treatment of diabetes mellitus. In this study, a simple, rapid, and effective method for enzyme inhibitors screening assay was established based on α-glucosidase catalyzes reactions in a personal glucose meter (PGM). α-glucosidase catalyzes the hydrolysis of maltose to produce glucose, which triggers the reduction of ferricyanide (K_3_[Fe(CN)_6_]) to ferrocyanide (K_4_[Fe(CN)_6_]) and generates the PGM detectable signals. When the α-glucosidase inhibitor (such as acarbose) is added, the yield of glucose and the readout of PGM decreased accordingly. This method can achieve the direct determination of α-glucosidase activity by the PGM as simple as the blood glucose tests. Under the optimal experimental conditions, the developed method was applied to evaluate the inhibitory activity of thirty-four small-molecule compounds and eighteen medicinal plants extracts on α-glucosidase. The results exhibit that lithospermic acid (52.5 ± 3.0%) and protocatechualdehyde (36.8 ± 2.8%) have higher inhibitory activity than that of positive control acarbose (31.5 ± 2.5%) at the same final concentration of 5.0 mM. Besides, the lemon extract has a good inhibitory effect on α-glucosidase with a percentage of inhibition of 43.3 ± 3.5%. Finally, the binding sites and modes of four active small-molecule compounds to α-glucosidase were investigated by molecular docking analysis. These results indicate that the PGM method is feasible to screening inhibitors from natural products with simple and rapid operations.

## 1. Introduction

Diabetes (Diabetes mellitus, DM) is a major public health problem that affects millions of people. Diabetes causes about 1.5 million deaths per year worldwide [1]. It is estimated that the number of people suffering from this disease will reach 592 million by the year 2035 alongside the serious sequelae and side effects of synthetic antidiabetic drugs [2]. DM is a group of syndromes resulted from insufficient secretion or reduced function of insulin due to different causes, leading to abnormal metabolism of carbohydrates, fats, and proteins. It is mainly manifested by chronic hyperglycemia, accompanied by chronic lesions of multiple systems such as lipid, cardiovascular, nerve, skin, and eyes. [3]. There are two main categories of DM: insulin-dependent diabetes mellitus (IDDM, type 1 diabetes) and non-insulin-dependent diabetes mellitus (NIDDM, type 2 diabetes) [4]. Particularly, type 2 diabetes is the main type of diabetes, which is mainly caused by glucose metabolism disturbances and the increase in blood glucose concentration due to the loss of insulin production by pancreatic β cells [5]. Besides, the decrease of postprandial hyperglycemia plays an important role in the treatment of diabetes. Because postprandial hyperglycemia is the first symptom in the onset of diabetes, which can induce various complications and lead to high mortality of diabetic patients. Therefore, as a crucial enzyme regulating postprandial blood glucose, α-glucosidase is often used as an important target for the treatment of type 2 diabetes [6,7,8]. While the appropriate inhibitors regulating the activity of α-glucosidase can achieve the purpose of treating diabetes. However, there are only a few α-glucosidase inhibitors (acarbose, voglibose, and miglitol) have been used in clinical practice. Furthermore, there still exist gastrointestinal adverse reactions of these inhibitors such as abdominal discomfort, flatulence, gastrointestinal spasm pain, and refractory constipation [9,10]. Thus, many researchers have begun to screen α-glucosidase inhibitors from natural products along with synthetic chemicals to find safe and effective new drugs.

Traditional analytical methods for detecting α-glucosidase activity include chromatographic [11,12], fluorescence [13,14], and colorimetric [15,16] methods, which usually suffer from the disadvantages of time and reagent consuming, requiring professional operators and expensive instruments. Although they are commonly applied in many studies, they are not feasible for point-of-care testing (POCT) and hinder their general application in resource restrained areas. Therefore, it is necessary to develop analytical methods for medical and environmental applications, which can achieve rapid determination without advanced equipment or complex operation.

**Scheme 1:**
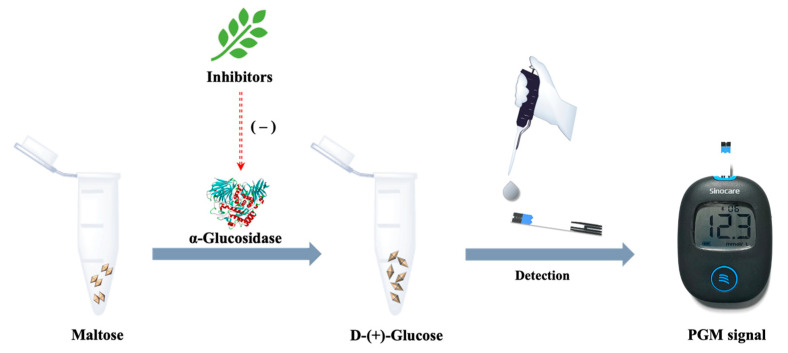
Graphic illustration of the principle of the PGM method based on α-glucosidase catalyzes the hydrolysis of maltose reaction.

As point-of-care diagnostic tools, personal glucose meters (PGMs) are well-placed to be repurposed for on-site and real-time molecule detection owing to their portability, low cost, simplicity, inherent quantification, and high selectivity [17]. Furthermore, the use of PGMs for the detection of biomarkers other than glucose are also extensively developed, such as enzymes [18], mycotoxins [19], metal ions [20], protein, and DNA [21,22]. Most published methods use enzymes or nanoparticles containing glucose to produce glucose that is ultimately measured by PGM. In our previous studies, β-glucosidase [23] and alkaline phosphatase [24] activity assays and their inhibitor screening methods have been successfully established using PGM. These two strategies are based on the target enzymes that can hydrolyze substrates (D(-)-Salicin or amifostine) to produce glucose and saligenin or generate thiol compound to trigger reduction of K_3_[Fe(CN)_6_], which can be detected by the PGM. In this study, a direct, sensitive, and efficient PGM-based method was developed for α-glucosidase inhibitor screening, which depends on the hydrolysis of one molecule of maltose to two molecules of glucose that is catalyzed by α-glucosidase. As shown in Scheme 1, the hydrolysis products of maltose (glucose) trigger the reduction of K_3_[Fe(CN)_6_] to K_4_[Fe(CN)_6_]. Then, K_4_[Fe(CN)_6_] can be re-oxidized at the electrode surface in the glucose strips to generate the microcurrent. PGM detects micro currents and converts them into glucose concentrations. When the α-glucosidase inhibitor (such as acarbose) is added, its inhibition on α-glucosidase will decrease the yield of products and reduce the readout of PGM. Therefore, α-glucosidase inhibitor screening assay can be achieved without any complex procedure. The proposed PGM method was used to evaluate the inhibitory activity of thirty-four small-molecule compounds and eighteen medicinal plants extracts on α-glucosidase, and the binding sites and modes of four active small-molecule compounds to α-glucosidase were predicted by molecular docking study.

## 2. Results and Discussion

### 2.1. Principle of the PGM Method Based on α-Glucosidase-Mediated Cascade Reaction

Scheme 1 illustrates the principle of the PGM method of α-glucosidase inhibitory activity assay. Glucose is produced by the hydrolysis of maltose, which is catalyzed by the α-glucosidase. The glucose triggers the reduction of K_3_[Fe(CN)_6_] to K_4_[Fe(CN)_6_] and generates the PGM detectable microcurrent. The addition of inhibitors could reduce the yield of glucose and decrease the readout of the PGM. Based on this principle, α-glucosidase inhibitors screening assay can be performed.

**Figure 1:**
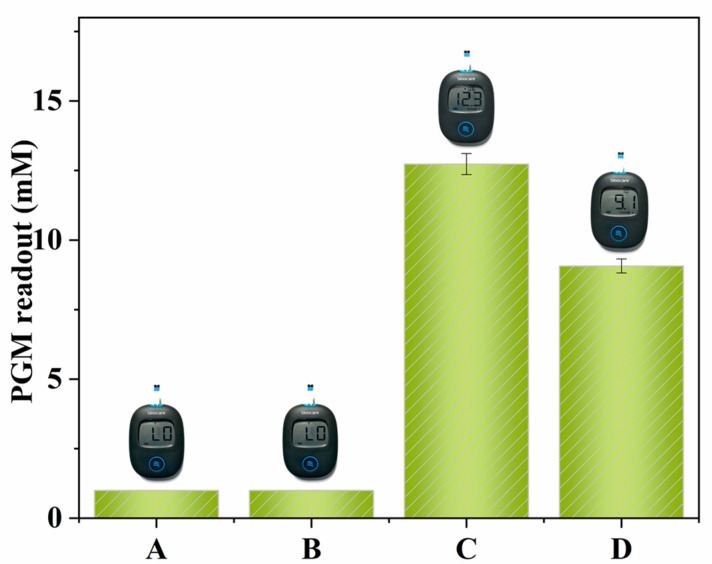
The PGM readout of different solutions based on α-glucosidase catalyzes the reaction. (**A**): Maltose solution; (**B**): α-Glucosidase + acarbose hydrate; (**C**): α-Glucosidase + maltose solution; (**D**): α-Glucosidase + maltose solution + acarbose hydrate; L0: PGM readout < 1.1 mM. Conditions: α-glucosidase (enzyme activity ≥ 7 × 10^5^ U/mL), 10.0 mM maltose solution, 0.13 mM acarbose hydrate, and inhibitor incubation and enzymatic reaction time were 5.0 min, respectively. All concentrations mentioned above are the final concentrations.

To evaluate the feasibility of the design principle, four solutions were measured by PGM. As shown in Figure 1, the PGM readout of the maltose solution or α-glucosidase + acarbose hydrate (Figure 1A or Figure 1B) are all L0 (L0 value means PGM readout < 1.1 mM). However, the PGM readout of α-glucosidase + maltose solution incubated for 5.0 min is 12.3 mM (Figure 1C). Furthermore, when the α-glucosidase inhibitor (acarbose hydrate) was added to the enzymatic reaction mixtures solution and incubated for 5.0 min, the PGM readout is decreased to 9.1 mM (Figure 1D). These results indicate that the PGM method is feasible to evaluate the α-glucosidase activity and screen enzyme inhibitors by analyzing the change of PGM readout.

### 2.2. Optimization of the Experimental Conditions

To confirm the optimal conditions for α-glucosidase, the experimental parameters, including pH values (2.0–7.0) of buffer solution, incubation temperature (40.0–60.0 °C), the relationship between PGM readout and final maltose concentration (2.0–12.0 mM), final maltose concentration (3.0–9.0 mM) and incubation time (4.0–8.0 min), were investigated.

**Figure 2:**
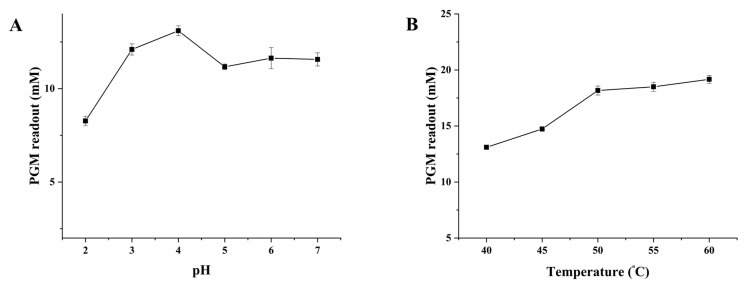
Effect of pH (**A**) of buffer solution and incubation temperature (**B**) on the PGM readout. Conditions: α-glucosidase (enzyme activity ≥ 7 × 10^5^ U/mL), 10.0 mM maltose solution and incubation time of 5.0 min. All concentrations mentioned above are the final concentrations.

As shown in Figure 2A, the PGM readout increases with the increase in pH, until the pH value at 4.0, the PGM readout reaches a maximum value and decreases from pH 5.0 to 7.0. Thus, phosphate-buffered saline (PBS, 10.0 mM) at pH 4.0 was selected for the subsequent experiments.

The temperature has an important impact on the α-glucosidase activity. As shown in Figure 2B, the PGM readout increases with the increase in incubation temperature, however, the PGM readout gradually levels off when the temperature reaches 50 °C. Thus, 50 °C was selected as the optimal incubation temperature for subsequent experiments.

**Figure 3:**
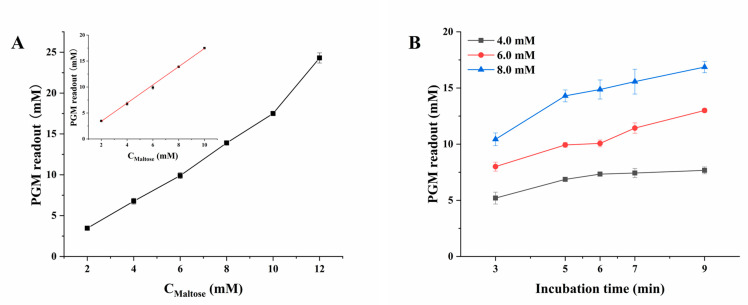
The concentration of maltose-dependence on the PGM readout (**A**). The inset is the linear relationship between the concentration of maltose and the PGM readout. Effect of incubation time and the concentrations of maltose (**B**) on the PGM readout. Conditions: α-glucosidase (enzyme activity ≥ 7 × 10^5^ U/mL), 2.0–12.0 mM maltose solution incubation at 50 °C for 5.0 min (**A**). α-Glucosidase of original concentration, 4.0–8.0 mM maltose solution incubation at 50 °C for 3.0–9.0 min (**B**). All concentrations mentioned above are the final concentrations.

Figure 3A shows the relationship between PGM readout and maltose concentration, the PGM readout vs. final maltose concentration exhibits excellent linear in a range of 2.0–10.0 mM, and the regression equation of PGM readout = 1.7530 × C_maltose_ − 0.1074 (R^2^ = 0.9992). These results demonstrate that the PGM-based method for the detection of products of α-glucosidase catalyzes reaction is feasible. Therefore, a final maltose concentration range from 2.0 to 10.0 mM was used for the subsequent experiments.

As shown in Figure 3B, the enzymatic catalyzes reaction can generate enough products for PGM readout in the final concentrations of maltose from 4.0 to 8.0 mM after incubation at 50 °C for 3.0 min. Furthermore, the PGM readout in all the concentrations of maltose gradually stabilized after incubation at 50 °C for 5.0 min. In addition, when the final concentration of maltose was 8.0 mM, the PGM readout reached a maximum value in the linear range. Therefore, the incubation time of 5.0 min and the final maltose concentration of 8.0 mM were selected for further study.

### 2.3. The Screening of Inhibitors of α-Glucosidase Using the PGM Method

The repeatability of the developed method was investigated based on the RSD% of the PGM readouts. All the detection were repeated in triplicate under the optimized conditions: α-glucosidase (enzyme activity ≥ 7 × 10^5^ U/mL), final maltose concentration of 8.0 mM incubation at 50 °C for 5.0 min. The run-to-run repeatability of the PGM readout using a batch (*n* = 5) of glucose strips was estimated as 1.4%. The repeatability of the PGM readout using different batches (*n* = 5) of glucose strips was estimated at 1.9%. Besides, the Z’ factor value was calculated as 0.93 (*n* = 8) according to Equation (3). These results indicated that this method can be applied to practical α-glucosidase inhibitor screening assay with good precision and reliability.

**Figure 4:**
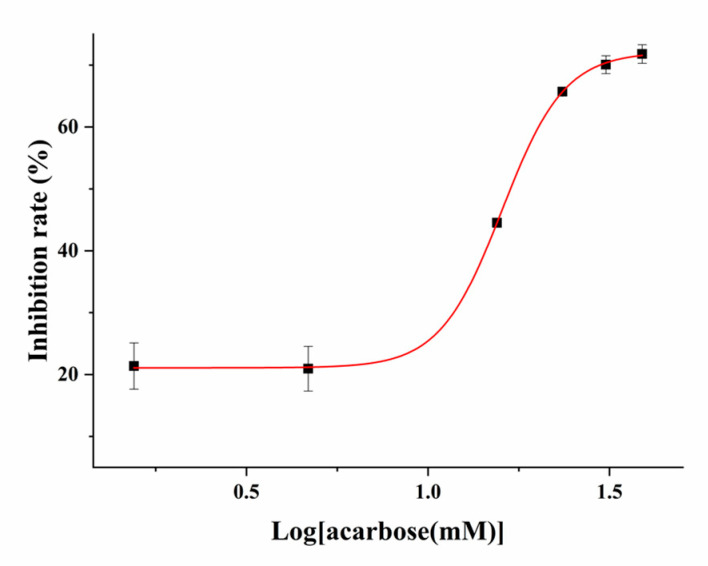
The inhibition curve for acarbose on α-glucosidase. Conditions: α-glucosidase (enzyme activity ≥ 7 × 10^5^ U/mL), 8.0 mM maltose solution, 1.6–46.5 mM acarbose solution, incubation temperature of 50 °C, inhibitor incubation and enzymatic reaction time were 5.0 min, respectively. All concentrations mentioned above are the final concentrations.

The inhibition plot of acarbose on α-glucosidase was shown in Figure 4. The IC_50_ value was calculated to be 16.8 mM through constructing a dose-response nonlinear regression equation. Furthermore, the inhibition activity of thirty-four small-molecule compounds on α-glucosidase were investigated at the final concentrations of 5.0 mM, and their percentage of inhibition results were shown in Table 1. Among these small-molecule compounds, phenolics show a stronger inhibitory activity, such as lithospermic acid and protocatechualdehyde exhibit a good inhibitory effect on α-glucosidase with the percentage of inhibition of 52.5% and 36.8%, respectively, which are higher than that of the positive control acarbose (percentage of inhibition of 31.5%). The 2,3,5,4′-tetrahydroxy stilbene-2-*O*-β-d-glucoside (TSG) shows comparable activities with acarbose, and its percentage of inhibition is 27.0%. Moreover, the percentage of inhibition of anhydrous citric acid, salvianolic acid B, and vanillic acid are around 20%, and the other compounds are considered to have no significant inhibitory effect at this concentration. In addition, the developed method was used to investigate the inhibitory activity of eighteen medicinal plants extracts on α-glucosidase at the concentrations of 0.2 mg/mL (Table 2). The results show that lemon has excellent inhibition efficacy and the percentage of inhibition is 43.3%. It was reported that polyphenols could modulate starch digestibility to reduce the glycemic, and the hydroxyl group and double bond in the structure of polyphenols play an important role in α-glucosidase inhibition [25]. These results indicate that the inhibitory effect of potential inhibitors on α-glucosidase can be determined by the PGM-based method.

### 2.4. Molecular Docking Study

**Figure 5:**
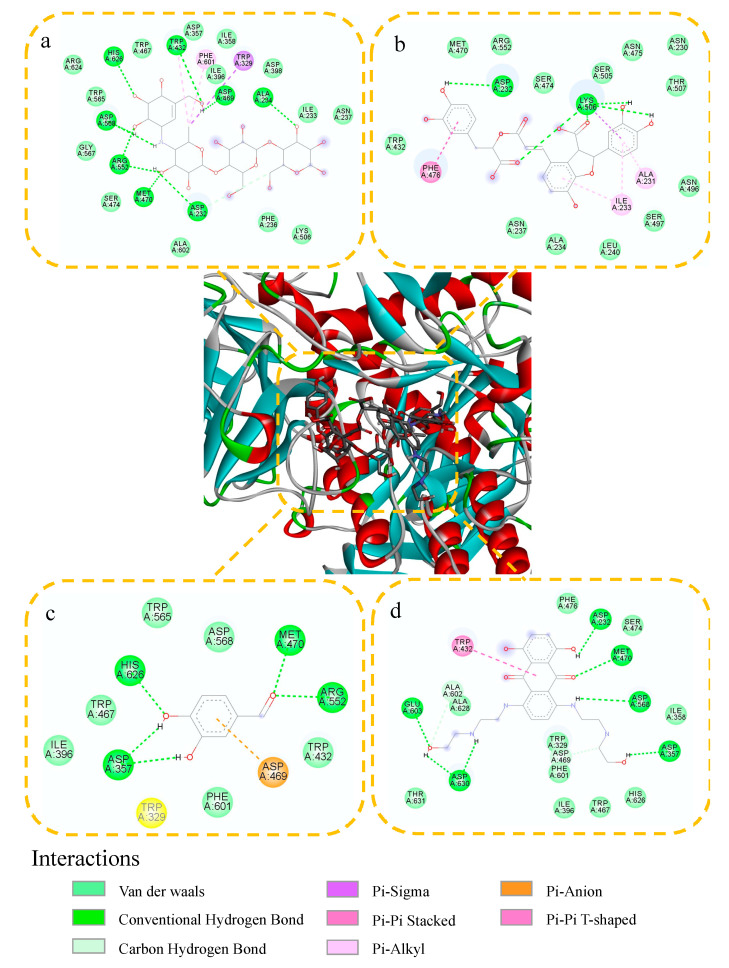
Molecular docking of acarbose (**a**), lithospermic acid (**b**), protocatechualdehyde (**c**), and TSG (**d**) with α-glucosidase.

To elucidate the interactions between α-glucosidase and the potential inhibitors, acarbose, lithospermic acid, protocatechualdehyde and TSG were selected as active compounds for the molecular docking study. The interaction diagrams of the best-docked conformations are shown in Figure 5, and the binding energy, hydrogen bonds, and amino acid residues of the interaction between α-glucosidase and the compounds are presented in Table 3. The results of the molecular docking study show that acarbose, lithospermic acid, protocatechualdehyde, and TSG are all located in the active site cavity of α-glucosidase and their binding energies are low. Besides, hydrogen bonds play an important role in the binding of ligands to α-glucosidase that exist between acarbose and ASP568, ASP232, ARG552, ASP469, ALA234, MET470, HIS626, TRP432; lithospermic acid and ASP232, LYS506; Protocatechualdehyde and ASP357, ARG552, HIS626, MET470; as well as TSG and GLU603, ASP630, ASP357, ASP232, ASP568, MET470. Compare with the positive control acarbose, these three compounds all have some similar amino acid residues. Particularly, ASP232, ASP357, HIS626, and MET470 are the most frequent amino acid residues which may be the important residues for the interactions between ligands and α-glucosidase. Moreover, the compounds containing hydroxyl, carbonyl, and amidogen groups, especially phenolic hydroxyl groups, are vital for hydrogen bond formation with α-glucosidase. This result corresponds to the result of inhibitor screening, and these compounds may be effective inhibitors.

## 3. Materials and Methods

### 3.1. Chemicals and Materials

The α-Glucosidase (liquid, enzyme activity ≥ 7 × 10^5^ U/mL) from aspergillus niger, protocatechuic acid, brucine, berberine hydrochloride, 2,3,5,4′-tetrahydroxy stilbene-2-*O*-β-d-glucoside (TSG), and theophylline were purchased from Shanghai Yuanye Biological Technology Co., Ltd. (Shanghai, China). Maltose solution was purchased from Shanghai Titan Scientific Co., Ltd. (Shanghai, China). Acarbose hydrate, tannic acid and 2,3,5,6-tetramethylpyrazine, gallic acid monohydrate, and chlorogenic acid were purchased from Shanghai Aladdin Biochemical Technology Co., Ltd. (Shanghai, China). Salvianolic acid B, salvianolic acid A, danshensu, protocatechualdehyde, lithospermic acid, puerarin 6’’-O-xyloside, syringin, and rosmarinic acid were purchased from Chengdu PureChem-Standard Co., Ltd. (Chengdu, China). Mogroside V, anhydrous citric acid, cryptochlorogenic acid, berbine, and paeoniflorin were purchased from Chengdu PUSH Bio-Technology Co., Ltd. (Chengdu, China). α-Arbutin was purchased from Shanghai Yien Chemical Technology Co., Ltd. (Shanghai, China). Catechin, gastrodin, vanillic acid, (-)-epigallocatechin gallate (EGCG), and (-)-epigallocatechin (EGC) were purchased from Chengdu Biopurify Phytochemicals Ltd. (Chengdu, China). (S)-(+)-Mandelic acid was purchased from Chengdu Aikoda Chemical Reagent Co., Ltd. (Chengdu, China). Trans-cinnamaldehyde was purchased from Meryer Chemical Technology Co., Ltd. (Shanghai, China). Genipin was purchased from Dalian Meilun Biotechnology Co., Ltd. (Dalian, China). Vanillin, sorbic acid, potassium dihydrogen phosphate (KH2PO4), and Na_2_HPO_4_•12H_2_O were purchased from Chengdu Chron Chemicals Co., Ltd. (Chengdu, China). Lemon was purchased from Sichuan Living Pharmaceutical Co., Ltd. Besides, seventeen medicinal plants pieces were purchased from Kangmei Pharmaceutical Co., Ltd. (Guangdong, China). All the samples were deposited at the Pharmaceutical Engineering Laboratory in the School of Chemistry and Chemical Engineering, Chongqing University, Chongqing, China.

### 3.2. Instruments

The PGM of Sannuo (Safe-AQ Air), blood glucose test strips (glucose detection ranges: 1.1–33.3 mmol/L) were purchased from Sinocare Inc. (Hunan, China). A FE 28 pH meter (Mettler-Toledo Instruments, Shanghai, China) was used for measuring the pH of solutions. The ultrasonic cleaner was purchased from Kunshan Jielimei Ultrasonic Instrument Co., Ltd. (Jiangsu, China). A palm-type centrifuge LX-100 was purchased from Jiangsu Haimen Qilinbeier Instrument Manufacturing Co., Ltd. (Jiangsu, China). RHP-100 High-Speed Multifunctional Crusher was purchased from Yongkang Ronghao Industry and Trade Co., Ltd. (Zhejiang, China).

### 3.3. Preparation of Solutions and Samples

The phosphate-buffered saline solution (PBS, 10.0 mM) was prepared by dissolving Na_2_HPO_4_•12H_2_O-KH_2_PO_4_ in ultrapure water and the required pH was adjusted by concentrated H_3_PO_4_. Maltose solution (24.0 mM, pH = 4.0) was prepared by dissolving maltose in PBS buffer and protected from light. Small-molecule compounds and acarbose hydrate were prepared by dissolving them in 10.0 mM PBS (pH = 4.0) with a final concentration of 5.0 mM, respectively.

The medicinal plant extracts were prepared by boiling water extraction. In brief, 2.0 g dried medicinal plant samples powder (through an 80-mesh sieve, 0.18 mm) were added into test tubes containing 10.0 mL boiling (100 °C) ultra-pure water under ultrasonic extraction for 30.0 min at room temperature, respectively. After extraction, the mixture solution was centrifuged and then the supernatant was filtered through a 0.22-μm nylon membrane filter (Shanghai Titan Scientific Co., Ltd., Shanghai, China). The medicinal plant extract solutions were stored at 4 °C before use.

### 3.4. Analytical Procedure for α-Glucosidase Inhibitory Activity Evaluation

The enzymatic reaction catalyzed by α-glucosidase was carried out in a 1.0 μL of α-glucosidase (enzyme activity ≥ 7 × 10^5^ U/mL), 1.0 μL of PBS (10.0 mM, pH = 5.0) and 1.0 μL of maltose solution (24.0 mM). After being incubated at 50 °C for 5.0 min, a volume of 1.0 μL of the mixture was directly measured by the PGM.

For the α-glucosidase inhibition study, a 1.0 μL of α-glucosidase was pre-incubated at 50 °C for 5.0 min with various inhibitors before the enzymatic reaction. Subsequently,1.0 μL of maltose solution (24.0 mM) was added to initiate the enzymatic reaction. Furthermore, the solution without maltose was served as a background solution and measured by the PGM. In brief, 1.0 μL of small-molecule compounds solution (15.0 mM) or medicinal plant extracts (0.2 g/mL) was added to 1.0 μL of α-glucosidase, and then 1.0 μL of PBS (10.0 mM, pH = 5.0) solution was added and incubated at 50 °C for 5.0 min before measured by PGM.

### 3.5. Inhibition Kinetics Study and Validation of the PGM-Based Method

The acarbose is a typical α-glucosidase inhibitor, which was used as a model compound for evaluating the inhibition kinetics on α-glucosidase. IC_50_ was measured by changing the concentration of acarbose (1.6–46.5 mM) with a constant concentration (8.0 mM) of maltose. The percentage of inhibition (*I%*) can be calculated through Equation (1):

(1) $$I\left(\%\right)=\left(1-\frac{I_{t}-I_{b}}{I_{0}}\right)\times 100\%$$

where *I_t_* and *I_0_* are the PGM readout with and without the inhibitor, respectively. *I_b_* is the background readout. The inhibition plot was constructed by dose-response nonlinear regression equation using Origin 2018.

*Z’* factor is the main parameter to estimate the performance of the activity evaluation method, which is calculated through Equation (2):

(2) $$Z’=1-\frac{3\sigma_{s}+3\sigma_{c}}{\left|\mu_{s}-\mu_{c}\right|}$$

where *μ_s_* and *μ_c_* are the averages of the signals of standard (s) (without inhibition) and the negative (c) control (100% inhibition by the reference inhibitor), respectively. The *σ_s_* and *σ_c_* denote the standard deviations of the signal. When the inhibition ratio is 100 %, *μ_c_* is zero, and Equation (2) can be translated into Equation (3). When the value of the Z’ factor is greater than 0.5, the developed activity evaluation method is accurate and reliable.

(3) $$Z’=1-\frac{3\sigma_{s}}{\mu_{s}}$$

### 3.6. Molecular Docking Study 

The binding sites and modes of the potential inhibitors to α-glucosidase were investigated by molecular docking. The known crystal structure of α-glucosidase (PDB ID: 3W37) was downloaded from the Protein Data Bank [26]. Before docking, the water molecules and acarbose ligands in the crystal structure 3W37 were all deleted while polar hydrogen was added. Besides, the chemical structures of the potential inhibitors and acarbose were drawn by Chem Office 3D and minimized the energy. The grid box center values were set to 0.109, −1.917, and −23.053 along the X, Y, and Z axes, respectively, with 0.375 Å grid spacing, and size values were specified as X = 50, Y = 50 and Z = 50. After docking, the conformation with the lowest energy was selected for analysis and pictured by Discovery Studio.

## 4. Conclusions

In this study, a simple, rapid, and effective method for enzyme inhibitors screening was established based on α-glucosidase catalyzes the reaction in PGM. Compared with the other α-glucosidase inhibitor screening methods, the prominent merit of the developed PGM method for α-glucosidase inhibitory activity evaluation of small-molecule compounds or medicinal plant extracts are as simple as the glucose in blood tests without any complex processing steps. Moreover, this method can screen effective inhibitors from natural products in a short time. The results of the inhibition activity tests demonstrate that lithospermic acid, protocatechualdehyde, TSG, and lemon extract have a high inhibitory activity on α-glucosidase. The results of the molecular docking study suggest that hydrogen bond plays an important role in the binding of ligand to α-glucosidase. Particularly, the compounds containing phenolic hydroxyl group are vital for hydrogen bond formation with α-glucosidase. Based on these results, phenols are considered to be effective α-glucosidase inhibitors. In short, this study provides a portable approach for the POCT of α-glucosidase, which can be further applied in diet therapy, medical diagnostics, and environmental monitoring.

## Figures and Tables

**Table 1:** The percentage of inhibition of thirty-four small-molecule compounds on α-glucosidase was determined by the PGM method (*n* = 3).

Compounds	Inhibition (%)	Compounds	Inhibition (%)	Compounds	Inhibition (%)
Acarbose	31.5 ± 2.5	Protocatechuic acid	11.9 ± 2.6	Rosmarinic acid	0
Lithospermic acid	52.5 ± 3.0	Catechin	10.3 ± 4.2	(-)-Epigallocatechin gallate	0
Protocatechualdehyde	36.8 ± 2.8	Puerarin 6’’-O-xyloside	7.5 ± 6.8	Gallic acid monohydrate	0
2,3,5,4′-Tetrahydroxy stilbene-2-*O*-β-d-glucoside	27.0 ± 2.0	Cryptochlorogenic acid	4.6 ± 3.7	Genipin	0
Anhydrous citric acid	20.7 ± 3.4	Theophylline	4.5 ± 1.5	Gastrodin	0
Salvianolic acid B	19.4 ± 1.8	Sorbic acid	1.1 ± 1.4	Brucine	0
Vanillic acid	19.0 ± 1.8	Chlorogenic acid	0.9 ± 0	Berbine	0
Vanillin	17.6 ± 0.7	Berberine hydrochloride	0.9 ± 4.7	2,3,5,6-Tetramethylpyrazine	0
Danshensu	17.2 ± 0.9	α-Arbutin	0	trans-Cinnamaldehyde	0
(-)-Epigallocatechin	14.1 ± 1.7	Syringin	0	Tannic acid	- ^1^
(S)-(+)-Mandelic acid	13.6 ± 3.7	Mogroside V	0	-	-
Paeoniflorin	12.8 ± 1.6	Salvianolic acid A	0	-	-

^1^ The PGM readout of the background is too large to be measured.

**Table 2:** The percentage of inhibition of eighteen medicinal plant extracts on α-glucosidase was determined by the PGM method (*n* = 3).

Extracts	Inhibition (%)	Extracts	Inhibition (%)	Extracts	Inhibition (%)
Lemon	43.3 ± 3.5	Common Anemarrhena	5.1 ± 2.5	Radix Polygoni multiflori preparata	0
*Ligusticum chuanxiong* hort	14.1 ± 2.5	Gengen	4.1 ± 3.2	Paeoniae Radix Alba	0
Mulberry Leaf	8.6 ± 0.6	Ural licorice	3.8 ± 0.4	Rhubarb	0
*Salvia miltiorrhiza*	8.0 ± 1.9	*Polygonatum sibiricum*	2.8 ± 2.3	Radix angelicae sinensis	0
Membranous milkvetch root	6.9 ± 1.9	Radix lithospermi	0.6 ± 1.7	Honeysuckle flower	- ^1^
Cinnamon	6.5 ± 3.5	*Polygonum cuspidatum*	1.2 ± 3.1	Chinese nut-gall	- ^1^

^1^ The PGM readout of the background is too large to be measured.

**Table 3:** Docking results of investigated small-molecule compounds with α-glucosidase.

Compounds	Binding Energy (Kcal/mol)	Hydrogen Bonds	Other Amino Acid Residues
Acarbose	−5.03	ASP568, ASP232, ARG552, ASP469, ALA234, MET470, HIS626, TRP432	TRP467, TRP565, ARG624, ASP357, ILE358, ILE396, ASP398, ILE233, ASN237, LYS506, PHE236, LA602, SER474, GLY567, PHE601, TRP329
Lithospermic acid	−4.68	ASP232, LYS506	TRP432, MET470, ARG552, SER474, SER505, ASN475, ASN230, THR507, ASN496, SER497, LEU240, ALA234, ASN237, PHE476, ILE233, ALA231
Protocatechualdehyde	−4.96	ASP357, ARG552, HIS626, MET470	ILE396, TRP467, TRP565, ASP568, TRP432, PHE601, ASP469, TRP329
2,3,5,4′-Tetrahydroxy stilbene-2-O-β-D-glucoside	−8.74	GLU603, ASP630, ASP357, ASP232, ASP568, MET470	THR631, ALA602, ALA628, PHE476, SER474, ILE358, TRP329, ASP469, PHE601, ILE396, TRP467, HIS626, TRP432

## Data Availability

The data presented in this study are contained within the article.

## Sample Availability

Samples of the compounds are available from the authors.
